# Relationships Among Non-Functional Occlusal Habits, Temporomandibular Disorder Symptoms, and Skeletal Morphology in Patients with Dentofacial Deformities

**DOI:** 10.3390/jcm14238330

**Published:** 2025-11-24

**Authors:** Yurie Fukagawa, Kazuhiro Ooi, Sae Nishino, Yutaka Sasajima, Kosuke Ueki, Rei Jokaji, Yusuke Nakade, Hirokazu Okita, Tetsutaro Yahata, Shuichi Kawashiri

**Affiliations:** 1 Department of Oral and Maxillofacial Surgery, Graduate School of Medical Science, Kanazawa University, Kanazawa 920-8641, Japan; yuri0808fuka@gmail.com (Y.F.); shirokuma55snow@gmail.com (S.N.); sasajima29346@gmail.com (Y.S.); ksk.uk.nud@gmail.com (K.U.); r_johkaji@staff.kanazawa-u.ac.jp (R.J.); skawa@med.kanazawa-u.ac.jp (S.K.); 2 Department of Clinical Laboratory, Kanazawa University Hospital, Kanazawa 920-8641, Japan; nakadeyukeitu@gmail.com; 3 Department of Rehabilitation Medicine, Kanazawa University Hospital, Kanazawa 920-8641, Japan; okita@med.kanazawa-u.ac.jp (H.O.); yahata@med.kanazawa-u.ac.jp (T.Y.)

**Keywords:** non-functional oral habits, temporomandibular disorder, dentofacial deformity, mandibular asymmetry, unilateral chewing

## Abstract

**Background/Objectives:** Non-functional oral habits, such as unilateral chewing, bruxism, and clenching, may exacerbate temporomandibular disorder (TMD) symptoms and affect skeletal morphology in patients with dentofacial deformities. This study aimed to elucidate the relationships among these habits, TMD symptoms, and mandibular morphology, and to examine the association between non-functional habits and TMD symptom severity. **Methods:** A cross-sectional study was conducted in 141 patients with dentofacial deformities. At the initial consultation, participants completed a questionnaire assessing non-functional oral habits (unilateral chewing, bruxism, and clenching) and TMD symptoms, scored as follows: limited mouth opening (2 points), pain on opening (2 points), and joint sounds (1 point). Patients were stratified into three groups according to the number of habits (≥2, 1, none). Total TMD scores were compared among groups, and the relationship between unilateral chewing and mandibular asymmetry was analyzed. **Results**: Unilateral chewing was reported by 82 patients (58%), bruxism by 28 (20%), and clenching by 29 (21%). Mean TMD scores were 1.94 (range: 0–5) in patients with ≥2 habits, 1.50 (0–5) in those with one habit, and 0.86 (0–3) in those without habits. TMD symptoms were significantly more severe in patients with multiple habits (*p* < 0.05). Among patients with mandibular asymmetry, 41 of 56 (73%) reported unilateral chewing, which was significantly higher than in those without asymmetry (42 of 85; 49%) (*p* < 0.05). **Conclusions**: In patients with dentofacial deformities, multiple non-functional oral habits were associated with greater TMD symptom severity, and unilateral chewing was significantly associated with mandibular asymmetry.

## 1. Introduction

Oral habits include tongue thrusting, lip sucking, thumb sucking, mouth breathing, and resting the chin on the hand, among others. Non-functional occlusal habits have habitual or psychological stress and depression causes, and examples include teeth grinding, clenching, and partial chewing. Functional occlusal habits have functional occlusion or muscular causes, and examples include functional mandibular prognathism and tongue thrusting due to functional occlusion problems. Among these, non-functional occlusal habits such as bruxism, clenching, and unilateral chewing have been reported to be significantly associated with temporomandibular disorders (TMD) [1,2,3]. Bruxism and TMD have a 17% prevalence worldwide, with significant differences between continents. The average prevalence of TMD among bruxism patients was reported to be 63.5% [4]. Non-functional occlusal habits, particularly bruxism, involve parafunctional activities such as grinding and clenching of the teeth [5], and their associations with TMD symptoms have been demonstrated in several studies [5,6,7]. Parafunctional habits such as bruxism are known to increase the risk of painful TMD in adolescents and adults, both cumulatively and synergistically [8]. Sleep bruxism, for instance, has been implicated in myofascial pain, temporomandibular joint (TMJ) pain, and intra-articular pathologies such as disk displacement and joint sounds [9,10]. Moreover, the interaction between sleep and awake bruxism has been associated with an increased risk of painful TMD [11].

Unilateral chewing is also regarded as a non-functional occlusal habit, similar to bruxism. However, little is known about the prevalence and clinical implications of these habits in patients with dentofacial deformities. Patients with dentofacial deformities, particularly those with mandibular retrognathia, open bite, or mandibular asymmetry, have been reported to present with higher rates of TMD symptoms [12]. Clinically, many patients with dentofacial deformities appear to have a history of TMD symptoms, which motivated the present investigation.

It has been hypothesized that non-functional occlusal habits during the growth period may affect mandibular morphology [13] and contribute to the development of dentofacial deformities, in addition to degenerative changes such as osteoarthritis. We hypothesized that patients with multiple non-functional occlusal habits exhibit higher TMD symptom severity and more frequent mandibular asymmetry. Bruxism, characterized by rhythmic activity of the masticatory muscles, is centrally regulated but also influenced by peripheral and psychosocial factors, including stress and anxiety [14]. Therefore, centrally mediated parafunctional habits may induce TMD symptoms, which in turn could influence mandibular growth and morphology, potentially contributing to the development of dentofacial deformities. This investigation was conducted as a retrospective observational study using preoperative clinical records and imaging data collected between 2015 and 2023 at Kanazawa University Hospital.

The aim of this study was to clarify the associations between non-functional occlusal habits and TMD symptoms in patients with dentofacial deformities.

## 2. Patients and Methods

### 2.1. Inclusion Criteria

This was a retrospective observational study that analyzed existing clinical records obtained at the initial consultation. All data were collected during the preoperative evaluation period between 2015 and 2023. This study included 141 patients (35 males and 106 females) who underwent orthognathic surgery for dentofacial deformities at Kanazawa University Hospital. The mean age of the patients was 24 years (range, 15–58 years).

Inclusion criteria for orthognathic surgery are skeletal and functional correction. Regarding skeletal classification based on ANB angle: 15 patients had skeletal Class I (ANB 2–4°), 25 patients had skeletal Class II (ANB > 4°), and 101 patients had skeletal Class III (ANB < 2°). Among the 141 patients, 38 presented with mandibular asymmetry and 57 with open bite.

#### Exclusion Criteria

Patients were excluded if they had:

- a history of systemic abnormalities, facial trauma, juvenile idiopathic arthritis, or idiopathic condylar resorption
- congenital anomalies
- pituitary adenoma
- unwillingness to provide informed consent

### 2.2. Methods

#### 2.2.1. Questionnaire on Non-Functional Occlusal Habits and TMD Symptoms

A questionnaire was administered at the initial consultation.

The questionnaire used in this study was developed originally in our department as a simple screening tool to assess the presence of parafunctional habits and self-reported TMD symptoms during the initial consultation. Bruxism was assessed using a self-reported item: “*Do you grind your teeth?*”. This question was designed to capture the overall tendency toward bruxism-like behaviors without distinguishing between awake and sleep conditions. Because sleep bruxism requires objective diagnostic methods such as polysomnography or electromyographic recordings, which were not feasible in this retrospective clinical setting, a simplified self-report approach was used to ensure consistency across all participants. The severity of self-reported TMD symptoms was evaluated using a simple categorical scoring system: pain on opening and limitation of mouth opening were scored as 2 points each, and joint sounds as 1 point. This simplified scale was designed as a screening index rather than a detailed Likert-type assessment, allowing consistent application across all participants at the initial consultation.

The survey items included:

1. Presence of non-functional occlusal habits (during waking hours only; sleep-related behaviors were not considered):
  ○ unilateral chewing
  ○ bruxism
  ○ clenching
2. History of TMD symptoms, scored as follows:
  ○ limited mouth opening (2 points)
  ○ TMJ pain (2 points)
  ○ joint sounds (1 point).

#### 2.2.2. Evaluation of Skeletal Morphology

Skeletal classification (Class I, II, III), mandibular asymmetry, and open bite were evaluated using lateral cephalometric radiographs at the initial consultation. Skeletal classification was primarily determined based on the ANB angle, which reflects the relative anteroposterior relationship between the maxilla and the mandible. Participants with an ANB angle greater than 4° were classified as Skeletal Class II, and those with an ANB angle less than 2° were classified as Skeletal Class III, following previously established criteria [15]. To minimize possible misinterpretation due to mandibular rotation, SNA, SNB, and Wits appraisal were additionally evaluated as supporting parameters.

These indices were consistent with the ANB-based classification and confirmed the validity of the skeletal grouping used in this study. Mandibular asymmetry was defined as a menton deviation greater than 3 mm from the facial midline, measured on posteroanterior cephalometric radiographs. The facial midline was established as a perpendicular line drawn from the midpoint between the bilateral orbitale points to the menton. All cephalometric measurements were performed by one calibrated examiner with more than 10 years of experience in orthodontic and dentofacial analysis. To assess intra-examiner reliability, 20 randomly selected radiographs were re-measured after a two-week interval, and the intraclass correlation coefficient (ICC) exceeded 0.90 for all variables. For inter-examiner verification, a second examiner independently reviewed all measurements, and any discrepancy greater than 1.0 mm or 1.0° was jointly re-evaluated until consensus was reached. This standardized procedure ensured excellent measurement reproducibility.

#### 2.2.3. Statistical Analysis

Data were first tested for normality using the Shapiro–Wilk test. Patients were stratified into three groups according to the number of non-functional occlusal habits (≥2, 1, none). The total TMD symptom scores were compared among the three groups using a one-way ANOVA followed by Bonferroni’s post hoc test for multiple comparisons. Effect sizes for the ANOVA were reported as Partial Eta Squared (η^2^), with η^2^ = 0.01 indicating a small effect, η^2^ = 0.06 indicating a medium effect, and η^2^ = 0.14 indicating a large effect. In addition, the association between unilateral chewing and mandibular asymmetry was statistically analyzed using the Chi-square test. *p* < 0.05 was considered statistically significant. The effect size for the Chi-square test was calculated using Cramér’s V. All statistical analyses were performed using GraphPad Prism version 10 (GraphPad Software, San Diego, CA, USA).

These analyses ensured adequate statistical validity. A post hoc power analysis was performed using G*Power version 3.1.9.7 (Heinrich-Heine-University, Düsseldorf, Germany). For the one-way ANOVA comparing TMD scores among three groups, with a total sample size of n = 141, an alpha level of 0.05, and a medium effect size (f = 0.25), the statistical power (1 − β) was calculated as 0.92, indicating that the sample size was adequate to detect significant differences.

## 3. Results

**Figure 1:**
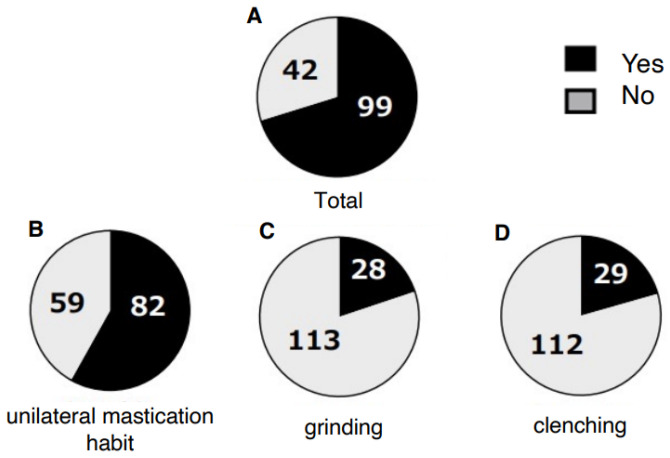
Subjective non-functional occlusal habits in patients with dentofacial deformities. The prevalence of unilateral mastication habit, grinding, and clenching is shown.

**Figure 2:**
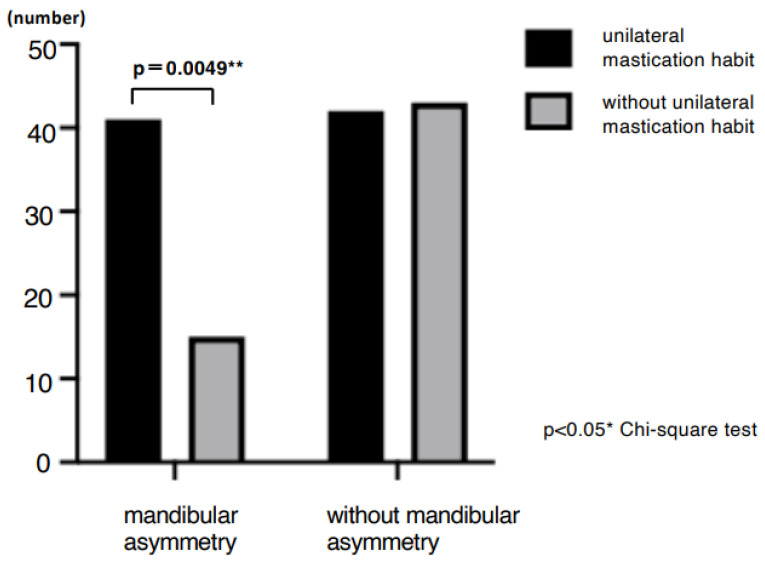
Relationship between unilateral mastication habit and mandibular asymmetry. The prevalence of unilateral mastication was significantly higher in patients with mandibular asymmetry than in those without (Chi-square test, * *p* < 0.05, ** *p* < 0.01).

**Figure 3:**
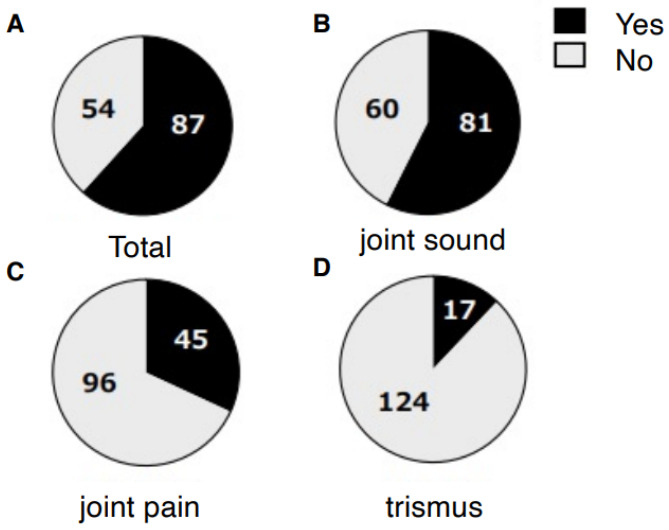
Subjective symptoms of temporomandibular disorder in patients with dentofacial deformities. The presence of joint sound, joint pain, and trismus is illustrated.

**Figure 4:**
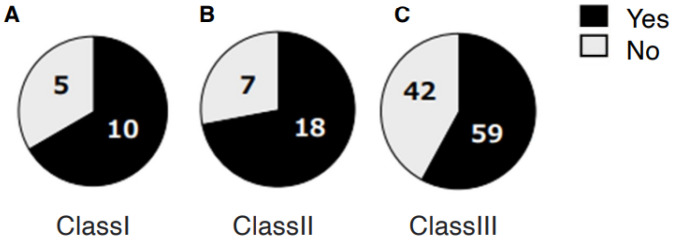
Total number of subjective temporomandibular disorder symptoms according to skeletal morphology. The prevalence of TMD symptoms is shown for skeletal Class I, II, and III patients.

**Figure 5:**
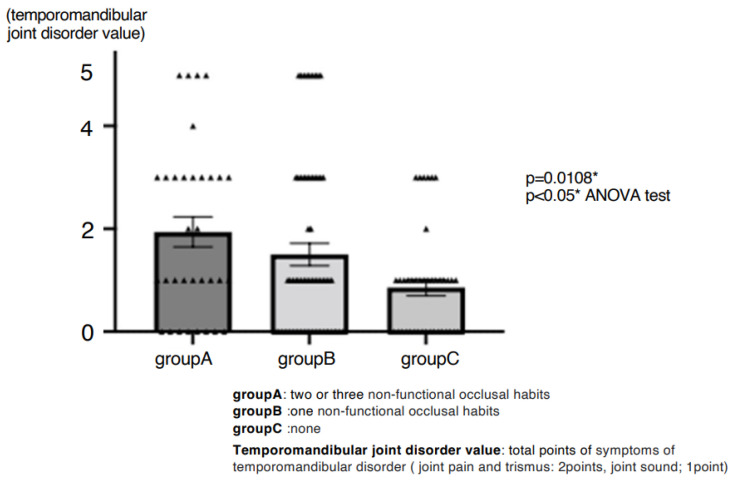
Relationship between non-functional occlusal habits and severity of temporomandibular disorder symptoms. TMD symptom scores (joint pain and trismus: 2 points each, joint sound: 1 point) were compared among patients with multiple NFOH (Group A), single NFOH (Group B), and none (Group C). Patients with multiple habits exhibited significantly higher TMD scores (ANOVA, * *p* < 0.05).

A total of 99 patients (70%) reported awareness of non-functional occlusal habits. Among them, unilateral chewing was reported by 82 patients (58%: 95% CI, 49.6–66.0), bruxism by 28 patients (20%: 95% CI, 13.5–27.0), and clenching by 29 patients (21%: 95% CI, 14.2–27.7) (Figure 1). Skeletal morphology according to sagittal classification and the presence of open bite are shown in Table 1; no significant differences were observed in these categories. However, non-functional habits were significantly more prevalent in patients with mandibular asymmetry, being present in 46 of 57 patients (81%) (*p* = 0.0249 Chi-square test, Cramér’s V = 0.22) (Table 2). Furthermore, unilateral chewing was significantly more frequent in patients with mandibular asymmetry (41 of 57; 72%) compared with those without asymmetry (42 of 85; 49%) (*p* = 0.0049 Chi-square test, Cramér’s V = 0.27) (Figure 2). Regarding TMD symptoms, 87 of 141 patients (62%) reported a history of TMD symptoms. Specifically, joint sounds were present in 81 patients (57%), TMJ pain in 45 patients (32%), and limited mouth opening in 17 patients (12%) (Figure 3). Thus, more than half of the patients with dentofacial deformities had a history of TMD symptoms. According to skeletal classification, TMD symptoms were observed in 10 of 15 Class I patients (66%), 18 of 25 Class II patients (72%), and 59 of 101 Class III patients (58%), indicating that TMD symptoms were more prevalent in Class II patients (*p* = 0.048, Chi-square test) (Figure 4). The mean TMD symptom scores were 1.94 (range, 0–5) in patients with two or more non-functional habits, 1.50 (0–5) in those with one habit, and 0.86 (0–3) in those without habits. TMD symptom severity was significantly higher in patients with multiple non-functional habits (*p* = 0.0108 ANOVA test, η^2^ = 0.07) (Figure 5).

## 4. Discussion

This study investigated the associations between non-functional occlusal habits and temporomandibular disorder (TMD) symptoms in patients with dentofacial deformities. The principal findings were as follows: (1) non-functional oral habits were reported by 70% of patients, with unilateral chewing being the most frequent; (2) unilateral chewing was significantly more common in patients with mandibular asymmetry; and (3) patients with multiple non-functional habits exhibited greater severity of TMD symptoms.

### 4.1. Prevalence and Impact of Parafunctional Habits

Our findings are consistent with epidemiological studies showing that awake bruxism has a prevalence of 23% in the general population [16], while sleep bruxism occurs in approximately 8% [17]. In the present cohort, bruxism and clenching were each observed in about 20% of patients, suggesting a higher frequency in dentofacial deformity patients compared to the general population. Bruxism, nail biting, and thumb sucking were found to be significantly associated with important oral/facial pain symptoms of clinical interest in the diagnoses of TMD indicating that those parafunctions are risk factors [18]. A significant association was observed between the presence of any dysfunctional oral habit and postoperative disk displacement with reduction [19].

Previous systematic reviews and clinical studies have consistently demonstrated significant associations between bruxism and TMD, including increased risk of myofascial pain, joint pain, and disk displacement [18,19]. Bruxer patients had more bony exostoses of the mandibular angle, smaller condyles, and morphological changes in cancellous and cortical mandibular bone compared to non-bruxer patients. Bruxism seems to induce morphological and anatomical changes in the different regions of the mandibular bone (condyles, mandibular angle, mandible body) [20]. Studies using CBCT and MRI have shown that patients with sleep bruxism have a higher prevalence of intervertebral disk deformation, intervertebral disk displacement, traumatic encephalopathy, and condylar bone changes [21].

CBCT revealed significant differences (*p* < 0.05) between patients with and without sleep bruxism in the right glenoid (AF)-axial plane (AP) and left glenoid-AP (B > NB), right submandibular angle (GA), left submandibular angle, saddle nose, and occlusal plane (B < NB), suggesting that bruxism may cause significant differences in the craniofacial morphology of the mandibular structure [13]. Furthermore, tooth contacting habit (TCH) has been identified as a behavioral factor prolonging chronic TMD pain [22]. Bruxism and dysfunctional oral habits were shown to be risk factors for the presence of TMD symptoms also after combined orthodontic and surgical treatment [19]. Importantly, preoperative parafunctional habits have also been shown to predict postoperative TMD symptoms even after orthognathic surgery [19], underscoring the importance of comprehensive functional assessment prior to treatment.

### 4.2. Unilateral Chewing and Mandibular Asymmetry

Unilateral chewing was associated with mandibular asymmetry in our patients.

It was reported that unilateral mastication might be an essential factor in the bilateral asymmetrical remodeling of the TMJ [23].

Population-based studies have demonstrated that chewing side preference is highly prevalent, with nearly half of individuals reporting a preference—most commonly for the right side [24]. Recent reviews have identified chewing side preference as a significant risk factor for TMD, with structural changes such as shorter condyles, increased articular eminence inclination, and altered fossa depth observed in patients with unilateral chewing [25].

During unilateral molar occlusion, mandibular deformity increases the pressure on the condyle and articular disk. Compared with mandibular protrusion or retraction, facial asymmetry will significantly increase the stress of the TMJ. Chewing on the non-deviated side will also lead to higher stress in the TMJ of patients with mandibular deviation [26]. As compared to the non-deviation side, the deviation side in the present class III patients with facial asymmetry showed a greater number of TMDs, such as condyle head deformity, disk displacement, and joint effusion [27].

Finite element stress analyses [26] and radiographic studies [27] have further confirmed that unilateral loading produces asymmetrical condylar deformation, disk displacement, and effusion, particularly on the deviated side. Clinical reports also show that patients with a unilateral chewing pattern present with significantly higher prevalence of TMD signs and symptoms [28]. Self-reported awake bruxism is highly associated with oral health-related quality of life in patients with temporomandibular joint osteoarthritis, while self-reported sleep bruxism and chewing-side preference are both moderately associated with oral health-related quality of life in patients with temporomandibular joint osteoarthritis [29].

Moreover, chewing-side preference and awake bruxism have been linked to reduced oral health-related quality of life in patients with TMJ osteoarthritis [29]. It has been suggested that pain caused by persistent clenching can cause damage to the articular disk, which in turn can lead to TMD [30]. Muscle asymmetry has been reported in patients with mandibular asymmetry [31], and muscle asymmetry has been reported to cause asymmetry of the condyle and result in morphological changes in the mandible [32,33]. It has been reported that the difference between the deflected and non-deflected condyles correlates with the development of facial asymmetry. CT scans of patients with mandibular asymmetry showed that the three-dimensional shape of the deflected condyle differed from that of the non-deflected condyle. This suggests a relationship between jaw asymmetry and joint remodeling [34]. It was reported that patients with TMD syndrome and unilateral chewing have abnormal changes in bilateral TMJ structure, showing medial and posterior displacement of the condyle on the unilateral chewing side and a compensatory increase in the pre-articular space on the non-unilateral chewing side [35]. Unilateral mastication affects the major compression sites in the left and right temporomandibular joints differently, suggesting that unilateral mastication may be an important factor in asymmetric remodeling of the TMJ [23]. A study using rats has shown that the condyles of growing humans become thinner while maintaining their length in the absence of occlusal stimulation [36]. It was reported that lateral imbalance of masseter muscle activity leads to inhibition of chondrogenesis and induces asymmetric formation of the condyle during the growth period [32]. In other words, this suggests that non-functional occlusal habits during the growth period cause asymmetry of the mandibular condyle and result in morphological changes in the mandible. These findings collectively support our observation that unilateral chewing is associated with both functional overload and skeletal asymmetry in dentofacial deformity patients.

### 4.3. Skeletal Morphology, Disk Displacement, and Joint Pathology

In addition to functional habits, skeletal morphology plays a crucial role in the pathophysiology of TMD. Previous studies have shown that skeletal Class II and hyperdivergent facial patterns are strongly associated with disk displacement and degenerative joint diseases [37]. When the temporomandibular joint disk displacement status was equal or bilaterally normal, the amount of mandibular deviation was not significant [38]. CBCT confirmed that a lesser condylar volume was also found on the deviation side in the asymmetric group. In the side with greater mandibular growth potential, the axis rotation in the axial plane would be greater [39].

In skeletal Class III patients with asymmetry, disk displacement has been found to correlate closely with mandibular deviation [38,39]. Our results, showing high prevalence of TMD symptoms in skeletal Class II patients, are in line with earlier reports by Ooi and colleagues, who demonstrated that anterior disk displacement without reduction (ADDwoR) was more frequently observed in skeletal Class III and asymmetric Class II patients [40], as well as in anterior open bite patients [41]. Similarly, Chou et al. [39]. confirmed that condylar morphology differs between deviated and non-deviated sides in Class III asymmetry, highlighting the structural basis for functional imbalance. Thus, both morphological and functional factors act synergistically in shaping TMD pathology in dentofacial deformity patients.

### 4.4. Psychosocial and Neurophysiological Considerations

The underlying mechanisms may involve both central and peripheral regulation of masticatory muscle activity. Parafunctional behaviors—especially those involving chronic muscle tension or abnormal mandibular positioning—may meaningfully contribute to the risk of TMD in high-stress student populations [42]. The inhibitory neurotransmitter gamma-aminobutyric acid (GABA) plays an important role in the pathophysiology of anxiety behavioral disorders such as panic disorder and post-traumatic stress disorder, suggesting that oral behaviors such as teeth grinding and clenching, generally known as bruxism, may be associated with disturbances in brain GABAergic and glutamatergic systems [43]. Recent neuroimaging studies on teeth grinding have revealed involvement of the hypothalamic–pituitary–adrenal (HPA) axis, which is also associated with temporomandibular disorder (TMD) and post-traumatic stress disorder (PTSD). Currently, teeth grinding, PTSD, and other stress-related mental disorders are thought to stem from dysfunction in circuits involving the medial prefrontal cortex/anterior cingulate cortex region, dorsolateral prefrontal cortex (DLPFC), hippocampus, and amygdala. The precise neurochemical mechanisms by which certain selective serotonin reuptake inhibitors (SSRIs) induce sleep bruxism, as well as mechanisms involving important co-factors such as sleep regulation, endocrine systems, autonomic nervous function, stress/anxiety, and motor control, remain a focus of research. The efficacy of drugs such as gabapentin, tiagabine, gamma-hydroxybutyric acid (GHB), diazepam, and lorazepam in improving bruxism suggests that gamma-aminobutyric acid (GABA), a major neurotransmitter, plays a significant role in bruxism [43]. Bruxism is influenced by psycho-social and behavioral factors, which means that oromandibular parafunctional activities, temporomandibular disorders, malocclusion, high levels of anxiety and stress, among others, may precipitate the occurrence of bruxism. It is currently linked to orofacial pain; headaches; sleep disorders; sleep breathing disorders, such as apnea and hypopnea sleep syndrome; behavior disorders, or those associated with the use of medications [14]. Based on the above, it appears that bruxism caused by stress, etc., may prolong disorders caused by bruxism, such as orofacial pain and headaches and sleep disorders. Multivariate analyses revealed that anxiety was associated with TMD pain, TMJ sounds, and combined TMD, while autonomy was related to TMJ sounds [44].

Patients with TMDs may have an upregulated hypothalamic–pituitary–adrenal axis with higher cortisol secretion from the adrenal cortex. Anxiety/depression and pain catastrophizing scores were significantly higher in the TMD group, and psychological factors may contribute to chronic upregulation of the hypothalamic–pituitary–adrenal axis [45]. Muscle activity was greater among individuals with severe temporomandibular disorder and positive correlations were found among electromyographic activity, salivary cortisol and the degree of temporomandibular disorder severity [46]. Hormonal and muscle function studies have also shown that temporomandibular joint disorder and stress are related [45,46].

After long-term unilateral mastication, changes in the stress within the joint cause the imbalance of temporomandibular joint (TMJ) structural reconstruction, the transformation and even destruction of the fiber structure of the masticatory muscle, resulting in uncoordinated movement of bilateral muscles. The joint neurogenic diseases caused by the increase in neuropeptide substance P and calcitonin-gene-related-peptide (CGRP) released locally by TMJ may be the mechanism of TMD [25]. Chronic parafunctional activity can lead to repetitive joint overloading, neuropeptide release, and structural remodeling [25], thereby exacerbating TMD symptoms and potentially influencing mandibular growth and asymmetry.

### 4.5. Clinical Implications and Treatment Outcomes

From a clinical perspective, more than half of the dentofacial deformity patients in this study presented with TMD symptoms, particularly those with skeletal Class II morphology. These findings are consistent with previous studies reporting high preoperative prevalence of TMD symptoms in orthognathic surgery candidates [47]. Importantly, orthognathic surgery has been shown to reduce TMD prevalence from 60.9% preoperatively to 34.4% one year postoperatively [47], with long-term improvements in pain and masticatory function [27]. However, a minority of patients may experience postoperative worsening [47]. Nonfunctional occlusal habits are risk factors for the development of TMD after surgery. Therefore, identifying nonfunctional occlusal habits before surgery and correcting these habits can prevent the development of TMD after surgery [19]. Methods for assessing parafunctional habits include subjective evaluations such as questionnaires regarding opinions on non-functional habits, and objective evaluations such as electromyography and ultrasound elastography of the masticatory muscles. Although congenital anomalies are excluded in this study, it is thought to be useful for screening diseases that include facial asymmetry and mandibular and maxillary retrusion as characteristic features, such as OFDI syndrome [48]. By identifying dysfunctional occlusal habits during the initial consultation and correcting these habits before surgery, we believe we can prevent the development of temporomandibular joint disorders after surgery.

### 4.6. Limitations

This study has several limitations. First, the cross-sectional design precludes causal inference between non-functional occlusal habits, temporomandibular disorder (TMD) symptoms, and skeletal morphology. Second, the evaluation relied on self-reported questionnaires, which may be subject to recall bias. Third, advanced imaging modalities such as MRI or CBCT were not employed, limiting direct assessment of intra-articular pathology. Although age, sex, deformity type, and the number of parafunctional habits may influence TMD symptoms, the present study focused on bivariate associations rather than causal inference. Although a significant association was found between unilateral chewing and mandibular asymmetry, this cross-sectional study cannot establish a causal relationship. Future prospective studies should employ multivariate models to confirm these associations. Because the sample was predominantly female, the generalizability of the findings may be limited, and future studies with a more balanced sex distribution are warranted. In addition, as the study population consisted exclusively of Japanese patients treated at a single institution, caution is required when generalizing the findings to other ethnic or demographic groups. This study evaluates the relationship between nonfunctional occlusal habits and temporomandibular joint disorders in patients with dentofacial deformities. Since the impact on postoperative treatment outcomes was not assessed, no post-surgical follow-up information is available. As this study was retrospective in design, the analysis was limited to information available in the existing clinical records, which may reduce control over potential confounding variables.

In the future, evaluating the efficacy of habit-modification programs, behavioral therapy, or biofeedback may help clarify whether early intervention before and after orthognathic surgery contributes to postoperative functional and symptom resolution. Furthermore, postoperative changes in TMD symptoms were not evaluated because the present study focused exclusively on preoperative data. Future longitudinal studies should assess postoperative outcomes to determine the influence of habitual behaviors on recovery.

### 4.7. Future Perspectives

Future research should employ longitudinal and interventional designs to elucidate causal pathways linking non-functional occlusal habits, TMD symptoms, and skeletal deformities. The integration of multimodal assessments, including MRI, CBCT, and ultrasound elastography, will enable objective evaluation of structural and functional alterations in the masticatory system. Moreover, studies assessing the efficacy of habit-modification programs, behavioral therapy, or biofeedback—particularly before and after orthognathic surgery—could help clarify whether early intervention improves postoperative function and symptom resolution. Finally, interdisciplinary investigations combining biomechanical modeling, neuromuscular physiology, and psychosocial assessment are expected to deepen our understanding of how habitual loading patterns interact with skeletal morphology and joint health. Future studies with larger sample sizes should include multivariate analyses to adjust for these potential confounding factors.

### 4.8. Summary of the Discussion

In summary, this study demonstrated that non-functional oral habits—particularly unilateral chewing and bruxism—are significantly associated with TMD symptom severity and mandibular asymmetry in patients with dentofacial deformities. The integration of functional habit evaluation with skeletal and joint assessments is essential for accurate diagnosis and comprehensive management of these patients.

## 5. Conclusions

This study demonstrated that non-functional oral habits, particularly unilateral chewing and bruxism, are highly prevalent among patients with dentofacial deformities and are significantly associated with the severity of temporomandibular disorder (TMD) symptoms and mandibular asymmetry. Patients with multiple parafunctional habits exhibited greater TMD symptom scores, and unilateral chewing was strongly related to asymmetric mandibular morphology. These findings suggest that oral habits may contribute to both functional overload of the temporomandibular joint and the development or exacerbation of skeletal asymmetry. From a clinical perspective, comprehensive assessment of non-functional habits should be considered an integral part of diagnosis and treatment planning in patients with dentofacial deformities. Identifying and addressing such habits preoperatively may improve management of TMD symptoms, optimize surgical outcomes, and reduce postoperative risks. Future longitudinal studies incorporating objective functional and imaging evaluations are warranted to clarify the causal pathways linking oral habits, TMD symptoms, and skeletal deformities.

## Figures and Tables

**Table 1:** Patients’ characteristics of dentofacial deformities.

		Open Bite/Asymmetry			Total
	(−/−)	(+/−)	(−/+)	(+/+)	
Skeletal class I	0	7	10	2	15
Skeletal class II	12	8	6	1	25
Skeletal class III	48	23	41	11	101

(number: patients).

**Table 2:** Relationship between non-functional occlusal habits and skeletal morphology of dentofacial deformity.

Class of Non-Functional Occlusal Habits		Skeletal Morphology of Dentofacial Deformity				
		Class III	Class II	Class I	Open Bite	Asymmetry
NFOH group	Group A	22	6	5	13	14
	Group B	46	12	8	16	32
non-NFOH group	Group C	33	7	2	9	11
*p* value		0.2338	0.8294	0.1405	0.3358	0.0249 *

Statistical analysis was performed between the NFOH group and the non-NFOH group. *p* < 0.05 * Chi-square test (number). Group A: two or three non-functional occlusal habits. Group B: one non-functional occlusal habit. Group C: none. NFOH: non-functional occlusal habits.

## Data Availability

The data supporting the findings of this study are available from the corresponding author upon reasonable request.
